# Association between Perfluoroalkyl substances and thyroid stimulating hormone among pregnant women: a cross-sectional study

**DOI:** 10.1186/1476-069X-12-76

**Published:** 2013-09-08

**Authors:** Yan Wang, Anne P Starling, Line S Haug, Merete Eggesbo, Georg Becher, Cathrine Thomsen, Gregory Travlos, Debra King, Jane A Hoppin, Walter J Rogan, Matthew P Longnecker

**Affiliations:** 1 Department of Health and Human Services, National Institute of Environmental Health Sciences, National Institutes of Health, Durham, NC, USA; 2 Department of Epidemiology, The University of North Carolina at Chapel Hill, Chapel Hill, NC, USA; 3 Department of Environmental Medicine, Norwegian Institute of Public Health, Oslo, Norway; 4 Department of Genes and Environment, Division of Epidemiology, Norwegian Institute of Public Health, Oslo, Norway; 5 Department of Chemistry, University of Oslo, Oslo, Norway

**Keywords:** Perfluorinated alkyl substances, Thyroid stimulating hormone, Pregnant women, The Norwegian Mother and child cohort study, MoBa

## Abstract

**Background:**

Perfluoroalkyl substances (PFASs) are a group of highly persistent chemicals that are widespread contaminants in wildlife and humans. Exposure to PFAS affects thyroid homeostasis in experimental animals and possibly in humans. The objective of this study was to examine the association between plasma concentrations of PFASs and thyroid stimulating hormone (TSH) among pregnant women.

**Methods:**

A total of 903 pregnant women who enrolled in the Norwegian Mother and Child Cohort Study from 2003 to 2004 were studied. Concentrations of thirteen PFASs and TSH were measured in plasma samples collected around the 18^th^ week of gestation. Linear regression models were used to evaluate associations between PFASs and TSH.

**Results:**

Among the thirteen PFASs, seven were detected in more than 60% of samples and perfluorooctane sulfonate (PFOS) had the highest concentrations (median, 12.8 ng/mL; inter-quartile range [IQR], 10.1 -16.5 ng/mL). The median TSH concentration was 3.5 (IQR, 2.4 - 4.8) μIU/mL. Pregnant women with higher PFOS had higher TSH levels. After adjustment, with each 1 ng/mL increase in PFOS concentration, there was a 0.8% (95% confidence interval: 0.1%, 1.6%) rise in TSH. The odds ratio of having an abnormally high TSH, however, was not increased, and other PFASs were unrelated to TSH.

**Conclusions:**

Our results suggest an association between PFOS and TSH in pregnant women that is small and may be of no clinical significance.

## Background

Perfluoroalkyl substances (PFASs) are a group of man-made chemicals with a fully fluorinated hydrophobic linear carbon chain attached to a hydrophilic functional group [1]. Being both lipo- and hydrophobic, they are widely used in industry to make products that are resistant to heat, oil, stains, grease and water [2]. Furthermore, the very high strength of the carbon-fluoride bonds makes PFASs highly stable and resistant to environmental degradation [3]. As a consequence, they are widespread in the environment, wildlife, and humans [4]. Diet is thought to be the main exposure route of PFASs for humans [5,6], and ingestion of dust from indoor environments may also contribute to the internal dose [7]. PFASs may act as endocrine disruptors by altering the activity of endogenous hormones [8].

Thyroid hormones play an essential role in ensuring a healthy pregnancy and normal fetal growth and development. Fetuses depend on the mother’s supply of T4 entirely during the first trimester, and continue to rely on it to varying degrees throughout the pregnancy [9,10]. At birth, around 30% of thyroid hormone is from the mother [11]. Alterations of maternal thyroid hormone status, even mild, are associated with adverse outcomes for the mother and offspring [12,13].

In pregnant rats and their offspring, treatment with perfluorooctane sulfonate (PFOS) can cause a decrease in total plasma thyroxine (T4) and triiodothyronin (T3) [14-17]. In humans, some PFASs have been associated with elevated T4 levels [18-20] and higher exposure to perfluorooctanoic acid (PFOA) or PFOS was related to increased risk of thyroid disease [21]. Other studies, however, did not find any association between PFASs and thyroid function [22-24]. Findings have generally been inconsistent and most studies were carried out in non-pregnant adults. One study on PFAS in pregnancy, however, showed an association of maternal pregnancy serum PFOA with increased cord TSH [25].

Given the potential association between PFASs and thyroid hormones, concern arises about whether PFAS exposure may disrupt maternal thyroid hormone homeostasis during pregnancy. In the present study, our aim was to examine the relationship between PFASs and TSH as a screening measure of thyroid dysfunction in pregnant women.

## Methods

### Study design

The Norwegian Mother and Child Cohort Study (MoBa) is a prospective population-based pregnancy cohort study conducted by the Norwegian Institute of Public Health [26]. Participants were recruited from all over Norway from 1999–2008, and 38.5% of invited women consented to participate. They received a postal invitation and the baseline questionnaire in weeks 13–17 of pregnancy, together with an appointment for an ultrasound scan in week 17–18 of pregnancy. The Regional Committee for Medical Research and the Norwegian Data Inspectorate approved the study, and signed informed consent was obtained from each participant.

The subjects in the present study were from a previous case–control analysis of subfecundity in a subset of MoBa women [27]. These subjects (N = 950) were selected from MoBa participants who enrolled in 2003 and 2004, delivered a live born child and provided a blood sample around week 17–18 of gestation. Among these eligible women, 400 subfecund women (who planned their pregnancy and reported time to pregnancy >12 months) were selected randomly. Additionally, 550 control women were selected at random. More details can be found in the previous publication [27]. Twenty of the 950 women reported a thyroid abnormality and were excluded from the main analyses.

### Assessment of PFASs

Blood specimens were collected at enrollment [28], and the mean gestational age at blood draw was 18 weeks. Up to thirteen PFASs were measured in plasma using high-performance liquid chromatography/tandem mass spectrometry at the Norwegian Institute of Public Health; these were PFOS, PFOA, perfluoroheptanoic acid (PFHpA), perfluorononanoic acid (PFNA), perfluorodecanoic acid (PFDA), perfluoroundecanoic acid (PFUnDA), perfluorododecanoic acid (PFDoDA), perfluorotridecanoic acid (PFTrDA), perfluorotetradecanoic acid (PFTeDA), perfluorobutanoic acid (PFBA), perfluorohexane sulfonic acid (PFHxS), perfluoroheptane sulfonic acid (PFHpS), and perfluorooctane sulfonamide (PFOSA). A detailed description of the analytic method has been published [29]. Among the substances measured, PFOA, PFOS, PFDA, PFHpS, PFHxS, PFNA, and PFUnDA were detected in more than 69% of samples; the six other PFASs were detected in less than 25% of the samples and were not included in the subsequent analysis. The limit of quantification (LOQ) was 0.1 ng/ml for PFBA and 0.05 ng/ml for all others. For the seven most frequently detected PFASs, values below the LOQ were replaced by the LOQ divided by the square root of 2. The intra-assay coefficients of variation (CVs) of all seven PFASs were < 10% except PFUnDA (13.1%), and the inter-assay CVs of all seven PFASs were < 15% except PFUnDA (18.7%) and PFHpS (25.2%).

### Assessment of TSH

Plasma TSH measurements were performed on the same plasma specimen using an immunoassay (MILLIPLEX map kit, Millipore Corporation, Billerica, MA, USA) on the LiquiChip 200 Workstation (Qiagen Inc., Germantown, MD) at the National Institute of Environmental Health Sciences, USA. Duplicate plasma TSH concentrations were measured for each sample and the mean was used in the analysis. The minimum detectable concentration was 0.01 μIU/mL. Both the intra-assay CV and inter-assay CV were <10%.

### Statistical analysis

The main outcome, TSH, was natural logarithm transformed (ln-transformed) to obtain a normal distribution. As there was no statistically significant difference in mean ln-TSH concentrations between the subfecund subjects and the control subjects (1.22 *vs* 1.19 μIU/mL, student t-test *P* = 0.34), we combined them in the subsequent analysis. Scatter plots showed approximately linear relationships between PFASs and ln-TSH, thus we used linear regression models to examine PFASs and ln-TSH and applied weighted methods (PROC SURVEYMEANS, PROC SURVEYREG and SURVEYLOGISTIC in SAS 9.2, Cary, NC, USA) to adjust for selection into the previous case–control study [30]. Each PFAS was examined in a separate model and was represented either as a continuous variable, or categorically based on quartiles. Logistic regression models were also used to examine TSH dichotomized as at or above the 95^th^ percentile (7.5 μIU/mL) or below.

We identified potentially confounding factors based on the literature [6,25,31,32]. They included age, gestational age at blood draw, pre-pregnancy body mass index (BMI, kg/m^2^), parity, smoking during pregnancy, interval between previous birth and current pregnancy, duration of breast-feeding a previous child, total seafood intake at mid-pregnancy, and plasma concentrations of high-density lipoprotein (HDL) and albumin. With the exception of age and gestational age, which were included *a priori*, confounders for inclusion in the models were identified using bivariate linear regression models with the exposure and outcome variables. Only those variables associated with any PFAS and also with TSH (*P* < 0.1) were included in the final models. Twenty-seven women were excluded because of missing data on covariates. Additional adjustment for consumption of fatty fish or use of medications that can affect thyroid hormone levels had essentially no effect on the results (not shown). In a sensitivity analysis we also fit a logistic model of self-reported thyroid abnormality during pregnancy (n = 20) or not (n =903) in relation to each PFAS. We also repeated the linear regression models of TSH after stratifying according to whether the women were in the control or subfecund group.

## Results

### Study population and plasma concentrations of PFASs and TSH

The average maternal age was 30 years (standard deviation [SD] = 4) and ranged from 18 to 44 years (Table 1). Almost all of the women (97%) were in the second trimester at the time of the blood draw (average, 18 weeks; range, 12–37 weeks). Half of the women were nulliparous.

**Table 1:** Characteristics of the 903 subjects

	**Mean (SD)**	**Range**
Age (yrs)	30 (4)	18 - 44
Gestational age (weeks)	18 (2)	12 - 37
Pre-pregnancy BMI (kg/m^2^)	25 (7)	15 – 45
HDL (mg/dL)	67 (12)	39 - 106
Total seafood intake (times/week)	5 (5.9)	0 - 56
Inter-pregnancy interval (months)	48 (37)	0 - 245
Duration of breast-feeding a previous child (months)	8 (6)	0 - 36
	%	
Parity		
Nulliparous	50	
Parous	50	
Smoking during pregnancy		
No	76	
Yes	24	
Maternal education		
< High school	9	
High school	33	
Some college	41	
≥ 4-year college	17	

Among the seven most frequently detected PFASs, the median concentration was greatest for PFOS (12.8 ng/mL; inter-quartile range [IQR], 10.1 -16.5 ng/mL), followed by PFOA, PFHxS, PFNA, PFUnDA, PFHpS, and PFDA (Table 2). Moderate positive correlations were found between concentrations of PFOS and PFOA (*r* = 0.66, *P* < 0.001), and between PFOS and PFHpS (*r* = 0.69, *P* < 0.001). The median blood concentration of TSH was 3.5 (IQR: 2.4, 4.8) μIU/mL.

**Table 2:** Distributions of plasma concentrations of PFASs (ng/mL) and TSH (μIU/mL)

PFASs ^*^	**% > LOQ**	**Geometric mean (95% CI)**	**25**^**th **^**percentile**	**Median**	**75**^**th **^**percentile**	**Maximum**
PFDA	69	0.09 (0.08, 0.09)	0.04	0.09	0.15	1.77
PFHpS	88	0.12 (0.11, 0.12)	0.08	0.13	0.18	0.97
PFHxS	99	0.62 (0.59, 0.64)	0.43	0.60	0.84	21.74
PFNA	99	0.37 (0.36, 0.39)	0.28	0.39	0.51	3.01
PFOA	100	2.13 (2.07, 2.20)	1.57	2.15	2.95	13.99
PFOS	100	12.77 (12.45, 13.10)	10.13	12.81	16.49	104.18
PFUnDA	94	0.20 (0.19, 0.21)	0.14	0.22	0.32	1.17
TSH	100	3.39 (3.25, 3.49)	2.44	3.52	4.81	18.64

* N = 903. For values of PFASs below LOQ, LOQs divided by square root of 2 were imputed and used in the calculations of statistics (PROC SURVEYMEANS).

Based on bivariate linear regression models, all PFAS concentrations in nulliparous women were higher than in parous women (*P* < 0.1). Among parous women, those who experienced longer inter-pregnancy interval had higher concentrations of PFASs, except PFHxS (*P* < 0.05). Most PFASs (all except PFHxS and PFOA) were positively associated with seafood intake in times/week (*P* < 0.05).

### Associations between PFASs and TSH

Among the seven PFASs, PFHpS, PFNA, and PFOS were positively associated with TSH levels (Table 3). Adjustment for confounders reduced the magnitude and statistical significance of associations between PFHpS and TSH, and PFNA and TSH. Only PFOS remained statistically significant after adjustment (*P* = 0.03), with a slight attenuation in the coefficient (β). To express the coefficients as percent change in TSH (original units), per ng/mL increase in PFOS, we calculated *100* × [*exp* (*β*) – *1*]. For example, the adjusted β of 0.008 for PFOS means a *100* × [*exp* (*0*.*008*) – *1*] or 0.8% (95% CI: [0.1%, 2.0%]) increase in TSH per ng/mL increase in PFOS.

**Table 3:** Unadjusted and adjusted β and 95% confidence intervals for associations between PFASs and TSH (ln-transformed)

**PFASs (ng/mL)**	**Unadjusted β (N = 903)**	Adjusted β (N = 903) ^*^
PFDA	0.246 (−0.258, 0.749)	0.060 (−0.458, 0.578)
PFHpS	0.537 (0.142, 0.932)	0.299 (−0.113, 0.710)
PFHxS	0.026 (−0.031, 0.084)	0.013 (−0.043, 0.070)
PFNA	0.251 (0.070, 0.432)	0.165 (−0.023, 0.353)
PFOA	0.029 (−0.008, 0.066)	−0.0001 (−0.045, 0.044)
PFOS	0.012 0.005, 0.019)	0.008 (0.001, 0.016)
PFUnDA	0.194 (−0.069, 0.457)	0.080 (−0.200, 0.360)

* Models adjusted for maternal age, gestational age at blood draw, HDL concentrations, total seafood intake, parity and inter-pregnancy interval.

**Figure 1:**
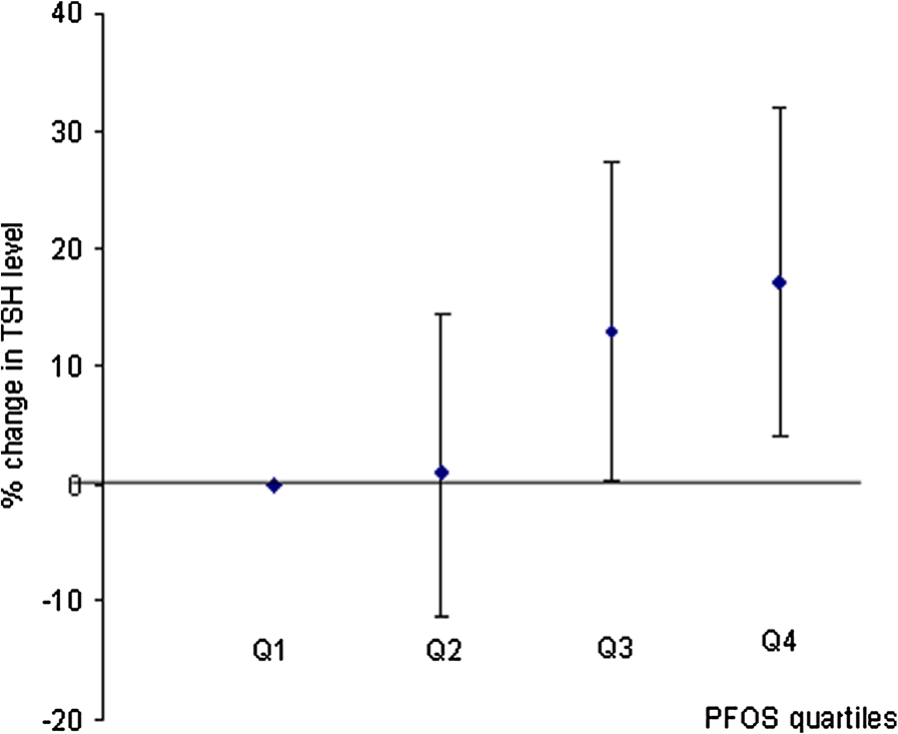
**Changes (%) in TSH level with increases in PFOS quartiles.** In the linear regression model, PFOS (ng/mL) was divided into quartiles (Q1: ≤ 10.30; Q2: >10.30 and ≤13.09; Q3: >13.09 and ≤16.58; Q4: >16.58). Q1 was used as reference group and each quartile was compared with the reference group. The model was adjusted for maternal age, gestational age at blood draw, HDL concentrations, total seafood intake, parity and inter-pregnancy interval.

Next, to examine the relationship between categorical PFASs and TSH levels we divided PFASs into quartiles and used the first quartile as the reference group. Figure 1 shows that there was an increasing trend in TSH across the quartiles of PFOS, but only women in the third and fourth quartiles had significantly higher TSH levels compared with the reference group.

When TSH was dichotomized (TSH ≥ 7.5 *vs* < 7.5 μIU/mL) in logistic models, no significant association with any PFAS emerged, whether the latter was considered either as a continuous or categorical variable. In addition, there were no significant associations found between PFASs and self-reported thyroid abnormality.

Among the women who were selected as controls from the MoBa cohort, we observed very similar results. The only significant association found was between PFOS and TSH (adjusted β = 0.008 [95% CI: 0.001, 0.016]). Among women in the subfecund group, the association was inverse and non-significant (β = −0.0016, 95% CI: [−0.008, 0.005]).

## Discussion

The precise classification of suboptimal thyroid status requires measurement of more than one hormone. For example, low free T4 with normal TSH reflects hypothyroxinemia [33]. Elevated TSH with normal free T4 reflects subclinical hypothyroidism. In the present study, where we used TSH by itself as a screening measure, we saw no indication that PFOS was associated with abnormally high TSH. Rather, the data suggested an association within the normal range of TSH, and in the absence of a measure of free T4, the clinical importance is unclear. Because we saw no association with abnormally high TSH nor with the presence of a history of thyroid disease, our generally null results were reassuring and consistent with a subtle association that may reflect only a biochemical curiosity. However, the possibility exists that our study did not have enough statistical power to detect a small association with abnormally high TSH, and that there is a small effect on public health (increased hypothyroidism).

In a study of 44 Korean pregnant women with serum measures of PFAS and several thyroid hormones in cord blood [25], inverse associations were found between maternal PFOS and neonatal total T3, and between maternal perfluorotridecanoic acid and neonatal total T3 and T4. In addition, maternal PFOA was associated with increased cord TSH (n = 31). However, the median maternal PFOS concentration (2.93 [IQR: 2.08, 4.36] ng/mL) was much lower than that in our study. In a case–control study of hypothyroxinemia among Canadian pregnant women (96 cases, 175 controls), the authors found no association with serum concentrations of PFOA, PFOS and PFHxS. In that study, the geometric mean of PFOS (7.39 ng/mL) [23] was lower than among women in the present study (12.77 ng/mL).

In animal experiments, treatment with PFAS generally does not affect serum TSH levels [14,34]. When effects on TSH are seen, they tend to be transient decreases after dosing [17]. PFASs appear to displace thyroxine from transthyretin, causing a transient increase in free T4 [35]. The end effect may be a reduced total T4 with adequate free T4 [14]. Data from animal models also indicate other potential mechanisms by which PFAS may affect thyroid hormone metabolism [34]. Thyroid metabolism in rodents, however, is different than in humans, partly because of transthyretin being a more important transport protein in rodents. Furthermore, during human pregnancy, human chorionic gonadotropin and estrogen affect the TSH feedback mechanism [36], further complicating comparison across species.

Compared with women from other western countries, the median concentrations of PFOS and PFOA in this study were slightly lower [21,37,38]. For TSH levels, the median in our study was higher than that in other studies of women in the second trimester [39,40]. The difference may explained by the use of different immuno-metric assays. TSH levels can differ by as much as 1.0 μIU/mL because different monoclonal antibodies may recognize different TSH isoforms circulating in the blood [36,41]. Iodine deficiency also affects TSH levels, but is rare in Norway [42]. The prevalence of self-reported thyroid disease in the present study (2.2%) was a little higher than that among pregnant women in another recent study from Norway [43].

We found concentrations of PFOA and PFHxS were both correlated with PFOS, suggesting they might come from common sources. However, they were not associated with TSH levels statistically and PFOA showed a different direction of association. These substances were usually present lower at levels than PFOS, and have different binding capacities with transthyretin due to different structures [35].

The observed association between PFOS and TSH was not present in the subfecund women. Although thyroid disorders are related to reduced fertility [44], we did not find a significant difference in TSH levels between subfecund women and controls. Subfecundity may mask the association, if any, between PFASs and TSH levels.

Our study had several limitations. Unfortunately, we did not have access to sufficient plasma volume to perform any other measurements of thyroid hormones, such as total T4, free T4 and T3. TSH, however, is a sensitive marker of thyroid status and reflects the physiologic log-linear relationship of TSH to free T4 [36], and is often used by itself in screening tests. Plasma TSH levels can reflect mild thyroid functional impairment even when plasma T4 and T3 concentrations are within normal ranges [45]. Second, because our study was cross-sectional study, reverse causation could affect the results. For example, if low thyroid function led to reduced glomerular filtration rate [46], this could increase circulating PFAS concentrations [47]. Third, as TSH levels change during pregnancy, a single measurement may not adequately characterize thyroid homeostasis during pregnancy. Fourth, although we considered some known potential confounders, there may be unmeasured confounders not taken into account. For example, the effects of other chemicals, such as polychlorinated biphenyls (PCBs), on TSH, can not be ruled out, given that fish intake is a source of both PFASs and PCBs. Finally, the participation rate in MoBa was low, raising the possibility of selection bias. Nilsen et al. compared general characteristics, exposures, pregnancy complications, and birth outcomes between MoBa participants and all women giving birth in Norway and found differences in prevalence estimates in most variables (Additional file 1: Table S1) [48]. For the subjects in the present study we tabulated their characteristics using the same format that Nilsen et al. used (Additional file 1: Table S1). The present subjects were generally similar to all MoBa participants, though some minor differences were apparent, as would be expected due to the subgroup sampled because of a long time-to-pregnancy. In spite of the differences between all women giving birth in Norway and MoBa participants, Nilsen et al. found estimates of exposure-outcome associations were similar in the whole population and the MoBa participants [48], and we expect this generalization holds for the present analysis, in which inverse-sampling weights were used.

## Conclusions

Our results suggest an association between PFOS and TSH in pregnant women that is small and maybe of no clinical significance.

## Abbreviations

BMI: Body mass index; CI: Confidence interval; CV: Coefficient of variation; HDL: High density lipoprotein; IQR: Inter-quartile range; LOQ: Limit of quantification; MoBa: The Norwegian Mother and child cohort study; PCBs: Polychlorinated biphenyls; PFASs: Perfluoroalkyl substances; PFBA: Perfluorobutanoic acid; PFDA: Perfluorodecanoic acid; PFDoDA: Perfluorododecanoic acid; PFHpA: Perfluoroheptanoic acid; PFHpS: Perfluoroheptane sulfonic acid; PFHxS: Perfluorohexane sulfonic acid; PFNA: Perfluorononanoic acid; PFOA: Perfluorooctanoic acid; PFOS: Perfluorooctane sulfonate; PFOSA: Perfluorooctane sulfonamide; PFTeDA: Perfluorotetradecanoic acid; PFTrDA: Perfluorotridecanoic acid; PFUnDA: Perfluoroundecanoic acid; T3: Triiodothyronine; T4: Thyroxine; TSH: Thyroid stimulating hormone.

## Competing interests

The authors declare that they have no competing interests.

## Authors’ contributions

MPL developed the study design and conducted the study; YW performed data analysis and drafted the manuscript; WJR and APS participated in reviewing, editing, and revising the manuscript; LSH, ME, GB, and CT contributed to the assessment of PFASs; GT and DK contributed to the assessment of TSH; and JAH assisted in the biobank of MoBa. All authors read and approved the final manuscript.

## Supplementary Material

Additional file 1: Table S1 Background characteristics, pregnancy-related exposures, and pregnancy complications [N(%)] for all women giving birth in Norway, MoBa participants, and subjects in the present study ^*^. Click here for file
