# Supplementary material for: Association between Perfluoroalkyl substances and thyroid stimulating hormone among pregnant women: a cross-sectional study
Source: Environ Health. 2013 Sep 8;12:76. doi: 10.1186/1476-069X-12-76 (PMC3847507; doi:10.1186/1476-069X-12-76)
Supplement: Additional file 1: Table S1 — Background characteristics, pregnancy-related exposures, and pregnancy complications [N(%)] for all women giving birth in Norway, MoBa participants, and subjects in the present study *. [file 1476-069X-12-76-S1.doc]

**Supplemental Table 1 Background Characteristics, Pregnancy-related Exposures, and Pregnancy Complications [N(%)] for All Women Giving Birth in Norway, MoBa Participants, and Subjects in the Present Study ^*^**

| **Background characteristics** | **Total population ^#^ (N=398849)** | **MoBa participants ^#^ (N=73579)** | **The present study ^†^ (M=903)** |
| --- | --- | --- | --- |
| Maternal age at delivery |  |  |  |
| <25 years | 67707 (17.0) | 8734 (11.9) | 74 (8.7) |
| 25-34 years | 266171 (66.7) | 52638 (71.5) | 687 (74.6) |
| >34 years | 64961 (16.3) | 12207 (16.6) | 162 (16.6) |
| Marital status |  |  |  |
| Single | 25547 (6.4) | 2599 (3.5) | 6 (0.7) |
| Cohabiting | 172287 (43.2) | 33930 (46.1) | 387(44.7) |
| Married | 195944 (49.1) | 36732 (49.9) | 506 (53.8) |
| Parity |  |  |  |
| 0 | 162983 (40.9) | 31763 (43.2) | 460 (44.6) |
| 1 | 142211 (35.7) | 26486 (36.0) | 323 (37.9) |
| 2 | 65770 (16.5) | 11840 (16.1) | 112 (13.6) |
| >2 | 27885 (7.0) | 3490 (4.7) | 28 (3.9) |
| Maternal asthma |  |  |  |
| Yes | 16468 (4.1) | 3155 (4.3) | 36 (4.1) |
| Maternal epilepsy |  |  |  |
| Yes | 3066 (0.77) | 554 (0.75) | 9 (1.3) |
| Pregestational diabetes |  |  |  |
| Yes | 2701 (0.68) | 415 (0.56) | 6 (0.15) |
| Smoking at the end of pregnancy |  |  |  |
| Unknown | 60254 (15.1) | 9512 (12.9) | 108 (14.6) |
| No | 295618 (74.1) | 59548 (80.9) | 750 (82.4) |
| Yes | 42977 (10.8) | 4519 (6.1) | 45 (3.0) |
| Multivitamin use before or any time during pregnancy |  |  |  |
| Unknown | 66443 (16.7) | 10310 (14.0) | 0 |
| No | 225628 (56.6) | 37391 (50.8) | 742 (61.5) |
| Yes | 106778 (26.8) | 25878 (35.2) | 161 (38.5) |
| Folic acid use before or any time during pregnancy |  |  |  |
| Unknown | 66443 (16.7) | 10310 (14.0) | 0 |
| No | 185974 (46.6) | 24551 (33.4) | 568 (42.7) |
| Yes | 146432 (36.7) | 38718 (52.6) | 335 (57.3) |
| Medication use during pregnancy |  |  |  |
| Yes | 85300 (21.4) | 17531 (23.8) | 224 (24.2) |
| Gestational diabetes |  |  |  |
| Yes | 3444 (0.86) | 587 (0.80) | 7 (0.76) |
| Preeclampsia |  |  |  |
| Yes | 15879 (4.0) | 2861 (3.9) | 40 (3.9) |
| Placental abruption |  |  |  |
| Yes | 1726 (0.43) | 282 (0.38) | 2 (0.20) |

* The data for the present study used here are from the Medical Birth of Norway as the total population and all MoBA participants.

# The data in the first two columns are from Nilsen et al. [49].

† Weighted percent by using surveyfreq procedure in SAS.
